# Management of critically ill patients in austere environments: good clinical practice by the Italian Society of Anesthesia, Analgesia, Resuscitation and Intensive Care (SIAARTI)

**DOI:** 10.1186/s44158-024-00209-8

**Published:** 2024-11-06

**Authors:** Mattia Bixio, Luca Carenzo, Giuseppe Accurso, Roberto Balagna, Simone Bazurro, Giovanni Chiarini, Andrea Cortegiani, Loredana Faraldi, Costantino Fontana, Emilio Giannarzia, Antonino Giarratano, Enrico Molineris, Santi Maurizio Raineri, Paolo Marin

**Affiliations:** 1 UO Anestesia E Rianimazione, IRCCS Ospedale Policlinico San Martino, Genoa, Italy; 2 Department of Anesthesia and Intensive Care Medicine, IRCCS Humanitas Research Hospital, Via Manzoni 56, Milan, Rozzano 20089 Italy; 3 UOC Anestesia Rianimazione E Terapia Intensiva, AOU Policlinico Paolo Giaccone, Palermo, Italy; 4 Anestesia e Rianimazione 2, Azienda Ospedaliero-Universitaria Città della Salute, Torino, Italy; 5 U.O. Anestesia E Rianimazione, Ospedale San Paolo, Savona, Italy; 6 Anestesia E Rianimazione, Spedali Civili Di Brescia, Brescia, Italy; 7 Dipartimento Di Discipline Di Medicina Di Precisione in Area Medica Chirurgica E Critica, Università Degli Studi Di Palermo, Palermo, Italy; 8 Servizio Anestesia E Rianimazione 1, ASST Grande Ospedale Metropolitano Niguarda, Milan, Italy; 9 Anestesia E Terapia Intensiva, Policlinico Militare, Rome, Italy; 10 Centro Ospedaliero Militare, Marina Militare, Taranto, Italy; 11 Anestesia E Rianimazione, Cuneo, ASL CN1 Italy; 12 Scuola Nazionale Medica del Soccorso Alpino (SNAMed), Corpo Nazionale Soccorso Alpino E Speleologico (CNSAS), Milan, Italy

**Keywords:** Austere, Hostile, SIAARTI, Critically ill patient, Critical care, Anesthesia, Airway management, Analgesia, Bleeding control, Vascular access, Medical devices and equipment

## Abstract

The Italian Society of Anesthesia, Analgesia, Resuscitation and Intensive Care (SIAARTI) has developed a good clinical practice to address the challenges of treating critically ill patients in resource-limited austere environments, exacerbated by recent pandemics, natural disasters, and conflicts. The methodological approach was based on a literature review and a modified Delphi method, which involved blind voting and consensus evaluation using a Likert scale. This process was conducted over two rounds of online voting. The document covers six critical topics: the overall impact of austere conditions on critical care, airway management, analgesia, bleeding control, vascular access, and medical devices and equipment. In these settings, it is vital to apply basic care techniques flexibly, focusing on immediate bleeding control, airway management, and hypothermia treatment to reduce mortality. For airway management, rapid sequence intubation with ketamine for sedation and muscle relaxation is suggested. Effective pain management involves a multimodal approach, including patient-controlled analgesia by quickly acting safe drugs, with an emphasis on ethical palliative care when other options are unavailable. Regarding hemorrhage, military-derived protocols like Tactical Combat Casualty Care significantly reduced mortality and influenced the development of civilian bleeding control devices. Establishing venous access is crucial, with intraosseous access as a swift option and central venous access for complex cases, ensuring aseptic conditions. Lastly, selecting medical equipment that matches the specific logistical and medical needs is essential, maintaining monitoring standards and considering advanced diagnostic tools like point-of-care ultrasounds. Finally, effective communication tools for coordination and telemedicine are also vital.

**Supplementary Information:**

The online version contains supplementary material available at 10.1186/s44158-024-00209-8.

## Background

Austere environments are characterized by extreme conditions and limited resources, including minimal infrastructure, scarce technological support, and challenging physical or logistical factors. These settings often present significant difficulties in delivering standard services, including healthcare, due to their harsh and resource-constrained nature [1]. Following a recent global pandemic, along with the increasing frequency of natural disasters and ongoing armed conflicts necessitating humanitarian efforts, there is a growing need to better understand the delivery of critical care medicine in austere, remote, and rapidly changing environments. The practice of austere critical care medicine stems from the understanding that logistical challenges in transporting supplies and patients to and from these challenging locations are just as vital as the medical care itself [2, 3]. Additionally, providing care in these environments demands specialized training. The Italian Society of Anesthesia, Analgesia, Resuscitation and Intensive Care (SIAARTI), recognizing the unique challenges of managing critically ill patients in austere environments, convened a group of content and methodology experts to develop a good clinical practice (GCP) document. The goal of this SIAARTI document is to support clinical decision-making in the care of critically ill patients in austere environments, based on a critical analysis of existing literature.

## Methods

The methodology behind this document is in line with the current SIAARTI regulations for the development of good clinical practice documents, including a consensus process [4].

### Scope and target audience

The scope of this document is to guide the care of critically ill patients in austere environments. The target audience includes physicians, critical care nurses, rescue personnel, and policymakers operating in the context of both high and low resources austere environments.

### Panel selection and organization

The SIAARTI Board of Directors selected the panel members based on their demonstrated clinical and scientific expertise. Diversity and inclusivity were key considerations in the selection process. The panel comprised ten experts, all practicing clinicians, from civilian and military backgrounds, including two coordinators (M.B. and P.M.). A methodologist (A.C.) oversaw the overall project.

### Items selections

The coordinators proposed six items which were then discussed during an online scoping workshop and prioritized through a voting process. The items were impact of austere environments on the delivery of medical care for critically ill patients, airway management, analgesia, bleeding control, vascular access, and devices and equipment. Different items were assigned to one or more panel members according to their respective expertise, to review the available literature and produce statements and supporting rationale in the form of explanatory text.

### Literature search

A systematic review of the literature was performed on each item, using both MeSH terms and keywords on PubMed. The search was restricted to articles published from 2000 ahead and in English. The types of articles included were meta-analyses, systematic reviews, narrative reviews, randomized controlled trials, original research papers, position papers, and guidelines. Articles not in English, conference proceedings, case reports, and case series were excluded. The search and reporting processes were carried out following the principles of PRISMA 2020 [5]. The search strategy is available as Electronic supplement A.

### Statements formulation

The panelists drafted statements and rationales using the literature and their own expertise, to then be put to a blind vote by the entire panel (excluding the methodologist). The complete list of statements was put to a vote following a modified Delphi process, to test the degree of consensus. The methodology used allowed for a maximum of two possible rounds of online voting. Opinions were expressed using an ordinal Likert scale, in accordance with the RAND/UCLA method. This consisted of an anonymous vote on an online platform where each panel member, after individually and collectively evaluating the literature available, was asked to express their judgment on appropriateness (appropriate, not appropriate, uncertain) by scoring each statement and rationale proposed from 1 to 9. Scores of 1–3 indicated inappropriate, 4–6 uncertain, and 7–9 appropriate. The Likert scale was divided into three sections: 1–3 implied rejection/disagreement (“not appropriate”); 4–6 implied “uncertainty”; and 7–9 implied agreement/support (“appropriate”) [6]. Voting members had the option to add comments or annotations as free text during the first round of voting. The consensus criteria used, established a priori, were as follows: (a) at least 75% of the respondents assigned a score of 1–3, 4–6, or 7–9, indicating rejection, uncertainty, or agreement and (b) the median score was within the same range. The type of consensus was determined by the positioning of the median.

The panel reached a consensus to develop the recommendations, and all members endorsed the final recommendations. It was not necessary to carry out a second round of voting, as all statements reached consensus. Four external reviewers (S.M.R., A.G., L.C., E.M.) reviewed the statements and provide feedback that were discussed with the panel and incorporated in the final version of the manuscript.

### Conflict of interest (COI) management

Panelists were required to disclose any intellectual or financial COI that could influence their involvement in the project by completing a specific form in accordance with SIAARTI procedures. Panelists did not receive any financial incentives for their participation. Furthermore, no funding or input from the industry was incorporated into the document. No COI were declared by the panelist.

## Results

### Impact of austere environments on the delivery of medical care for critically ill patients

Question:* Are there austere environments, situations, and conditions that hinder critically ill patient management and may worsen prognosis, where the application of appropriate and specific clinical practice is necessary to improve patient survival rates?*

Statements:

- Care for critically ill patients becomes challenging in environments outside of high resources settings, especially when there is significantly limited or no technological support and infrastructure.
- In austere environments, basic techniques and standardized protocols should be prioritized and applied flexibly based on the context to improve outcomes.
- In hostile settings, appropriate and timely management of massive hemorrhage, airway, and hypothermia may reduce mortality rate.

#### Background

Application of specific practices and protocols is crucial in austere environments, such as remote areas (including rural locations, deserts, high mountains, underground environments, ravines, open sea), during rescue operations in natural disasters (earthquakes, tsunamis, volcanic eruptions) and anthropogenic disasters (major accidents, industrial explosions, terrorist acts, war zones), in confined spaces, or else under adverse climate conditions (high altitude, arid regions, polar environments) and in rapidly evolving and unpredictable situations where operators face environmental hazards. From a medical perspective, these environments are often characterized by limited resources, challenging working conditions, and increased risks, which complicate the delivery of healthcare. If adequate actions are not taken, morbidity and mortality rates in these environments can be higher than in high resource settings [7].

Anesthesiologists and critical care specialists may intentionally or suddenly be involved in such environments, very different from their usual settings, often facing a sudden loss or severe limitation of technological support, medications, and infrastructures [8]. Managing critically ill patients in an austere environment is demanding and requires expertise, as well as dedicated training [9]. Considering that medical practice in austere environment often occurs in stressful scenarios with high risk of procedural errors, it is always preferable to follow standardized and simple working techniques [9]. Specific behavioral processes were developed in the second half of the nineties: first introduced in the military field and then later in civilian contexts. These allowed for a significant reduction in mortality and morbidity rates when implemented in austere environments. The main objectives in austere environments are rapid bleeding control (if present), safely airway management to promote adequate ventilation and oxygenation, maintenance of an adequate level of analgesia (which also aids reduce oxygen consumption), and vital organs protection [9–13].

### Airway management

Question: *Is rapid sequence intubation (RSI) the standard of care for airway management in critically ill patients requiring intubation in austere environments?*

Statements:

- RSI is widely recognized as an airway management procedure in hostile environments.
- There is a level of agreement in the literature regarding the drugs used for RSI, mostly on the role of ketamine as a sedative and analgesic. Moreover, muscle relaxation is crucial and fundamental for successful orotracheal intubation.
- Laryngeal manipulation maneuvers such as BURP (backward, upward, rightward pressure) can also be considered in hostile environments, in order to increase the possibility of successful RSI.
- Procedures like cricothyroidotomy and use of supraglottic devices represent valid alternatives for airway management, especially in patients who cannot be ventilated or intubated.
- When assessing the best technique for airway management, consideration must be given to the skill level of the operator, the feasibility of the maneuver, the nature of the environment, and the sustainability over time of the selected device, as well as the availability of devices and equipment.

#### Background

For patients requiring definitive airway control, RSI is the most widely recognized and preferable approach for airway management in austere environments. Alternatives are represented by positioning of supraglottic devices or emergency cricothyroidotomy (front-of-neck access procedure) [14–18]. Careful consideration must be given to the specific nature of the austere environment, such as climate conditions, terrain elevation, the presence of dangers for patients and healthcare professionals, and the availability of adequate resources (oxygen therapy, lung ventilator, monitoring, venous access, etc.), together with the possibility of evacuation and methods of transport, in order to choose the most appropriate method of airway management [18–21]. Cricothyroidotomy may be considered for airway management in hostile environments, especially if the patient cannot be ventilated or intubated; it is therefore preferable for practitioners who may be involved in such environments to be properly trained in this procedure [22–25]. Laryngeal manipulation maneuvers to improve first-pass success such as BURP (backward, upward, rightward pressure) are described in the approach to airway management in hostile environments. These techniques can improve tracheal visualization during laryngoscopy and increase the success rate of RSI [26, 27]. Several authors recommended pharmacological induction and paralysis to perform successful airway management in hostile environments. There is also agreement on the recommended drugs, with particular emphasis on ketamine as a sedative and analgesic due to its minimal hemodynamic impact. Similarly, adequate muscle relaxation is deemed essential and is strongly associated with the success of RSI [28–30]. Finally, there is a statistical correlation between the skills and experience of the healthcare professional performing the procedure and the success of RSI. Comparative studies on the success of RSI based on the operator performing the procedure suggest how success rates are statistically higher for specialists who have received adequate training [21, 23, 26, 27, 31]. To complement this rationale, we propose a schematic outline of a possible structured approach to airway management (Electronic Supplement B), based on 8 points (preparation, preoxygenation, priming, prime, pressure, paralysis, positioning, postintubation).

### Analgesia

Question: *What are the recommended approaches for managing pain in critically ill patients within austere environments?*

Statements:

- Pain is a critical vital sign that should be systematically assessed using validated scales. A multimodal and stepwise approach to pain management is preferable, focusing on treating both acute and procedural pain while also preventing the onset of chronic pain and post-traumatic stress disorder (PTSD). Whenever feasible, patient-controlled analgesia should be encouraged. Special care should be given to minimize the risk of narcotic substance abuse.
- Pain management is a priority of care, particularly in austere environments, considering the nature of potential injuries (such as multiple traumas, burns, or gunshot wounds) and the necessity of painful life-saving procedures (including immobilization, fracture reduction, wound dressing or compression, tourniquet application, and pneumothorax drainage). Additionally, it must address the possibility of extended transport or field care before hospitalization.
- The panel agrees that effective pain management enhances and improves the overall treatment of critically ill patients in austere environments, particularly when administered by a healthcare professional with specialized skills.
- Preferred drugs should fulfill the following characteristics: rapid onset, ease of titration, a broad therapeutic index, availability of specific antidotes, compatibility with other medications, and, if necessary, multiple routes of administration. They should also have minimal impact on hemodynamic stability and airway protection reflexes, while preserving respiratory drive, especially given potential limitations in monitoring and assistance.
- The panel also agrees on the fundamental humanitarian requirement of delivering effective and competent palliative care when injuries and operating conditions prevent the use of alternative treatments, ensuring strict adherence to ethical and medical-legal guidelines.

#### Background

Pain is the most frequently reported symptom for which medical assistance is sought, even in austere environments. It requires effective and timely treatment tailored to the specific environmental context, the patient’s injuries, life-saving maneuvers, and rescue methods [32–43]. Pain is considered the fifth vital sign, and the Numeric Rating Scale (NRS) is the most readily available tool for measuring and recording it alongside the administered therapy [38, 39, 44]. Moderate pain is indicated by an NRS score of 4 to 6, while severe pain is represented by a score of 7 to 10.

Pain is linked to significant stress responses, such as fever, tachycardia, tachypnea, hypertension, ileus, hypercoagulability, protein catabolism, and immunosuppression, and profoundly affects quality of life [45, 46]. This includes an increased risk of long-term morbidity and disability. Additionally, PTSD is strongly associated with a high prevalence of chronic pain [47–50].

Stepwise pharmacological approach commonly recommended in both civilian and military contexts suggests starting treatment with paracetamol or other oral non-steroidal anti-inflammatory drugs (including self-administration kits). For moderate to severe pain, in the absence of hemodynamic instability or respiratory distress, and when intravenous access is not available, oral transmucosal fentanyl or nasal fentanyl/ketamine is advised. These methods are preferred over intramuscular opiates, such as morphine, due to their slower onset and variable absorption rates, which increase the risk of overdose. When intravenous infusion is feasible, fentanyl, morphine (though not endorsed by all guidelines), and ketamine are the preferred options [40, 45, 51–58].

Locoregional anesthesia techniques require trained operators and specialized equipment. These techniques are most effective when applied quickly through a multimodal approach, particularly for managing pain from high-energy trauma to the extremities, where opioid monotherapy may not provide satisfactory relief [45, 52]. In hostile environments, the most described locoregional methods range from simple wound infiltration (short-term) to single-shot nerve blocks and the placement of perineural or epidural catheters for prolonged drug infusion. The latter options offer excellent efficacy, reduced opioid requirements, and lower rates of sedation or intubation, though their effectiveness largely depends on the operator’s expertise [59–62]. When selecting drugs for administration, it is crucial to account for the potential unavailability of monitoring equipment, oxygen sources, advanced airway management kits, fluids, and pressure support medications for various reasons [63, 64]. In more complex cases, an anesthesiologist or intensive care specialist or a pain specialist is best suited to implement multimodal therapy. This approach involves using multiple analgesics with different mechanisms of action in synergy to enhance overall pain control while striving to minimize the undesirable side effects of each drug [55].

Among opioids, fentanyl is the most suitable drug in hostile environment, despite growing concerns about its abuse, particularly in the USA [37, 39, 51, 55, 56, 65, 66]. Recent literature increasingly supports the use of ketamine for pain management in austere environments. At sub-anesthetic doses, ketamine provides analgesia that complements the effects of opioids, while at higher doses it produces dissociative anesthesia, effective pain relief, sedation, and amnesia [67–72]. Some studies suggest that ketamine may also enhance “resilience to stress” and help prevent the development of PTSD [50]. Ketamine can be combined with benzodiazepines or propofol to mitigate hallucinations [73]. Importantly, ketamine maintains hemodynamic stability and preserves spontaneous breathing and protective airway reflexes, even during extremely painful procedures [67–72]. The treatment of pain is a fundamental human right, as emphasized by numerous international conventions. However, this right may be compromised when healthcare resources are limited, such as during natural disasters, armed conflicts, or terrorist emergencies, which necessitate triage and resource rationing [74]. In such scenarios, anesthesiologists and critical care specialists operating in austere environments may need to employ palliative techniques when injuries and conditions prevent the use of alternative treatments [75–78].

### Bleeding

Question: *Can a prompt bleeding control contribute to improved survival rates?*

Statements:

- Time is a key factor in determining a favorable prognosis for patients with massive hemorrhage.
- Profuse and uncontrolled bleeding results in acute traumatic coagulopathy, a condition that poses a severe threat to the patient’s life.
- Coagulopathy, acidosis, and hypothermia commonly complicate massive hemorrhage, contributing to an increased mortality rate.
- Mortality is further increased if hemorrhage is also associated with hypocalcemia.

Question: *Are there established protocols for the timely control and management of bleeding, as well as suitable devices for the rapid control of hemorrhage at various anatomical sites?*

Statements:

- Protocols developed in military context, such as Tactical Combat Casualty Care, have led to a dramatic reduction in the mortality rate associated with massive hemorrhage.
- A tailored algorithm for civilian settings developed from military protocols significantly reduced mortality in austere environments.
- Insights from military medicine led to the development of devices for controlling hemorrhage in the upper and lower limbs.
- Following the effective use of these devices for controlling bleeding in limbs, additional devices have been developed to manage hemorrhage in non-compressible areas.

#### Background

Swift control of bleeding is a fundamental therapeutic measure for patient survival [13, 79]. Numerous studies support this statement, indicating that earlier intervention correlates with a lower mortality rate [79–84]. Timely management of bleeding, as outlined in protocols such as step X in X-ABCDE or M in MARCH (massive hemorrhage, airway, respirations, circulation, head injury/hypothermia: an acronym used by TCCC-trained individuals to help remember the proper order of treatment), is crucial not only as a major life saving intervention but also for preventing the development of the “lethal triad,” which comprises acute coagulopathy, acidosis, and hypothermia [80, 84–86]. In recent years, this “lethal triad” has been associated with a further pathological condition, linked to massive hemorrhage, entailing a negative prognosis: hypocalcemia. This tetrad is often referred to as the “Lethal Diamond” [87–89]. In 1996, a protocol was developed in the military setting emphasizing quick control of bleeding. The application of this protocol led to a significant reduction in mortality rates in operational environments [13]. In 2011, a version was published which is applicable for civilian environments [90].

These protocols agree that inspecting the patient for potential sources of bleeding should be the initial action. For a patient with external active bleeding, the first step is to perform manual compression at the bleeding site, which is a rapid and effective life-saving maneuver [91–96]. In cases of massive hemorrhage in the limbs, a tourniquet should be the primary device used, applied proximally to the injury. In military settings, it is advised to place the tourniquet at the top of the affected limb, where a single bone facilitates more effective arterial compression and minimizes the risk of movement during patient transfer. If applied correctly, the distal pulse should be absent; if not, a second tourniquet should be placed parallel to the first, a few centimeters away. Tourniquets should not be removed before reaching a healthcare facility or until a surgeon is present.

Continuous reassessment of the tourniquet is crucial, as several factors can cause bleeding to resume, including patient movement, displacement of the device, or removal and adjustment of the tourniquet by the patient due to severe pain. Adequate pain control is essential during this phase. For optimal bleeding control, compression and/or tourniquets should be combined with wound packing. It is important to pack the wound with hemostatic gauze by applying direct and continuous pressure, and then to bandage it with specific bandages [13]. In cases of bleeding in junctional areas (such as the groin or armpit) or in the head and torso, the use of hemostatic gauze for wound packing and a “combat” bandage is essential [13]. Junctional tourniquets for inguinal or axillary compression and abdominal tourniquets for managing lower body hemorrhages are available [97–101].

After controlling the bleeding, it is advisable to administer at least 1 g of tranexamic acid intravenously in cases of massive hemorrhage, as numerous studies following the CRASH-2 trial have underscored its significance in improving survival rates [102–105]. Calcium also plays a critical role in treating post-traumatic hemorrhage; recent research highlights the intravenous administration of calcium as a key step in managing trauma-induced coagulopathy [88, 89]. Early administration of empirical fibrinogen at a dose of 50 mg/kg can be considered in massive bleeding [106, 107]. Effective bleeding management must be complemented by proper body temperature control. Hypothermia, known as a silent killer in trauma patients, has been recognized as a critical component of the “lethal triad” since 1982 [108]. International guidelines recommend monitoring patient body temperature, using all available means to minimize heat loss, and implementing active warming strategies. Hypothermia is associated with a significant worsening in patient outcomes [109, 110].

### Vascular access

Question: *Are there specific recommendations for establishing venous access in austere environments and what type of access is preferable?*

Statements:

- When managing patients in austere environments, especially those in critical condition or at risk of rapid clinical deterioration, establishing adequate venous access is desirable. The decision to place venous access must be carefully evaluated based on feasibility, timing, and sustainability, considering the specific challenges of the specific austere setting.
- Intraosseous access is a suitable alternative to peripheral venous access in hostile environments. The panel does not endorse a specific intraosseous access device, as various devices are available. According to the literature, the recommended sites for intraosseous access include the humerus, tibia, and sternum. This method provides a quick and straightforward approach for infusing fluids, blood products, and medications.
- While central venous access is generally not recommended as a first choice in hostile environments, it may be considered for critically ill patients with specific therapeutic needs (such as those who are exsanguinating or require multiple infusions or medications that can only be administered via this route), provided it can be placed under aseptic conditions.

#### Background

Fluid resuscitation is universally recommended in the literature, particularly in traumatic scenarios occurring in hostile environments, to prevent or treat various manifestations of shock [111]. Obtaining adequate, rapid, and safe intravenous or intraosseous access is therefore essential [112, 113]. Timing for venous access placement should be carefully assessed based on the necessity of the procedure, patient’s condition, environmental factors, and any potential risks associated with the environment. Alternative routes for drug administration, such as intranasal, oral, or inhalation methods, should also be considered depending on the specific context. Ideally, venous access should be established in a stable location that remains accessible during patient maneuvers and transport.

Peripheral venous access can be challenging to establish due to frequent extremity injuries in trauma patients in extreme environments—whether tactical or other settings—compounded by adverse environmental conditions and technical difficulties. In contrast, intraosseous (IO) devices offer a rapid and straightforward solution for venous access in a variety of cases. Cooper et al. report a 97% success rate for intraosseous access placement in both hospital and tactical settings, demonstrating its efficacy for infusing various fluids, blood products, and medications, including those needed for RSI [112]. In tactical scenarios, IO access may even be preferable and associated with fewer complications compared to traditional peripheral venous access. When selecting an IO site, sternal or anterior tibial tubercle access has traditionally been preferred. In military and tactical contexts, the use of ballistic protective equipment and the high incidence of lower limb injuries (e.g., 60% of cases observed during the war in Afghanistan) have shifted preference towards the sternum as the primary access site. However, there are specific contraindications for sternal access, including suspected sternal fractures, a history of sternotomy, and situations involving cardiac arrest that necessitate external chest compressions [114]. Contraindications for tibial access include suspected fractures of the tibia or adjacent bone segments, as well as limb amputation. In certain traumatic scenarios, particularly in military settings involving blast injuries from improvised explosive devices (IEDs) with associated thoracic and lower extremity trauma, placing access in the humerus, potentially bilaterally, may be advantageous [115, 116]. IO accesses, regardless of the location, must in any case be considered temporary and ideally removed/replaced within 24 h of placement [113]. Central venous access provides a reliable and secure source of vascular access, but it is rarely used and generally not feasible in hostile environments, whether military or civilian, especially as a first choice [112, 117]. High-flow vascular catheters, often referred to as “trauma lines,” are employed in some military and civilian contexts. These catheters facilitate the rapid administration of large volumes of blood products, which is critical for managing exsanguinating hemorrhagic shock [118].

### Devices and equipment

Question: *What equipment should a physician working in an austere environment carry, and what characteristics should this equipment have?*

Statement:

- While a universal list of equipment and devices cannot be established, it is crucial to ensure that the equipment is tailored to the logistical constraints and is suitable for addressing critical issues, specific injuries, and risks pertinent to the scenario in which the healthcare professional is operating. Additionally, considerations should include the cost–benefit ratio and the sustainability of the equipment over time.
- The expert panel agrees that devices should adhere to current regulations and criteria, ensuring they are simple to use and applicable across various settings, even under extreme resource constraints.
- It is beneficial to standardize and verify equipment using a checklist organized by the level of care required, beginning with first aid supplies and progressing to equipment for advanced and definitive treatment.
- Oxygen supply is a critical factor and should be thoroughly evaluated during the planning phase before intervention.
- Ideally, basic monitoring standards recommended in anesthesia and intensive care contexts (non-invasive blood pressure [NIBP], pulse oximetry [SpO2], electrocardiogram [EKG], continuous end tidal CO2 monitoring [EtCO2], and temperature) should be maintained even in austere environments.
- Diagnostic tools, including those utilizing artificial intelligence, are particularly promising in these settings, as they can provide early warning signs by assessing changes in recorded clinical parameters.
- It is advisable to increasingly adopt point-of-care ultrasounds (POCUS) as a critical diagnostic tool in austere environments, accompanied by standardized training.
- Effective communication tools are also recommended for coordinating with rescue services and for utilizing telemedicine or teleconsultation aids.

#### Background

A definitive list of devices and equipment cannot be universally established, as medical practice in austere environments must adapt to a wide range of contexts. Each situation requires a specific assessment of conditions, logistics, and the capabilities of available facilities, while aiming to deliver high-quality, sustainable care over time and effectively allocating resources [2, 73, 119, 120]. Ideally, the minimum necessary equipment should be determined based on the anticipated level of treatment required and predetermined scenarios of care. While scientific literature does not provide a universally applicable checklist for equipment used in austere environments, standardizing and verifying equipment with a checklist based on the level of care required—ranging from first aid supplies to definitive treatment tools—is still beneficial [121–126]. It is important to account for factors that can accelerate wear and increase the risk of equipment damage in scenarios with limited access to assistance and maintenance [127]. Both the available literature and panel experience suggest that preferred devices should meet criteria for reliability under extreme temperature and humidity conditions and exhibit robustness. Additionally, they should be easily transportable, considering factors such as bulk, weight, and the integration of electronic medical devices. Ease of use and maintenance, including the potential for repair through methods like 3D printing, are also crucial considerations [128–130]. A lack of continuous electricity impacts equipment planning, making alternative energy sources, such as solar power for recharging batteries, a necessary consideration [131, 132]. Oxygen supply is particularly critical and must be carefully evaluated from a logistical standpoint, given its potential difficulty of procurement in extreme environments. Therefore, where feasible, the use of oxygen concentrators is preferred [133–138]. The panel recommends that in hostile environments, oxygen supplementation should primarily be reserved for patients with respiratory distress, those requiring assisted ventilation, or situations where reaching a higher-level care facility will be significantly delayed. Studies have shown no survival benefit from administering supplemental oxygen in prehospital settings to trauma patients who do not need assisted ventilation or airway protection, particularly when transport times are brief [139]. Monitoring standards typically recommended for conventional anesthesia and intensive care settings should also be maintained in hostile environments. Despite the resource limitations often encountered in such settings, ensuring these standards is crucial for the effective management of critically ill patients. The literature increasingly supports the use of bedside diagnostic techniques, such as POCUS, due to the proliferation of increasingly compact and affordable devices over the past 20 years. These devices, comparable in size to a smartphone, are easy to transport and have longer battery life, making them well-suited for use in austere environments [140]. Although conducting research in hostile environments is challenging and there are only a few methodologically significant studies supporting the use of ultrasounds, the literature highlights the widespread use of this technique in wilderness medicine. It is applicable to various situations and can be performed easily by healthcare professionals who are not specialized in radiology [141–151]. Several studies have shown that the use of ultrasounds in the field has repeatedly influenced triage, the management of critical patients, and the decision-making process. This has led to optimized clinical evaluations and more appropriate allocation of resources [152–158]. The panel of experts believes that providing monitoring and diagnostic equipment in the field must be preceded by comprehensive training for the healthcare professionals involved [159–164]. This training should focus not only on relevant medical preparation but also on basic surgical skills and the development of technical and soft skills. Such training should be conducted through continuing education and simulations, both prior to deployment and during live scenarios. Additionally, where possible, the use of “telementoring” platforms with augmented reality should be considered to enhance training and support [165–169]. Telemedicine and telemonitoring can play a pivotal role in managing patients in hostile environments by facilitating the direct transmission of expertise from specialized hospitals to remote locations. The use of these technologies is justified where possible but is not essential, as their effectiveness is strictly dependent on reliable connectivity capacity. Importantly, these devices should not be seen as replacements for physician training, which remains the most crucial tool for working in extreme environments [170–173].

## Conclusions

In conclusion, this GCP document includes 32 statements based on available literature regarding the care of critically ill patients in austere environments. These statements specifically address airway management, analgesia, bleeding control, vascular access, and the selection of devices and equipment.

## Supplementary Information

Supplementary Material 1

Supplementary Material 2

## Data Availability

No datasets were generated or analysed during the current study.
