# Supplementary material for: Management of critically ill patients in austere environments: good clinical practice by the Italian Society of Anesthesia, Analgesia, Resuscitation and Intensive Care (SIAARTI)
Source: J Anesth Analg Crit Care. 2024 Nov 6;4:74. doi: 10.1186/s44158-024-00209-8 (PMC11542215; doi:10.1186/s44158-024-00209-8)
Supplement: Supplementary file 1 — Supplementary Material 1 [file 44158_2024_209_MOESM1_ESM.docx]

**Management of Critically Ill Patients in Austere Environments: Good Clinical Practice by the Italian Society of Anesthesia, Analgesia, Resuscitation and Intensive Care (SIAARTI)**

Appendix B - Search Strategy

This string combines all the partial strings used by the subgroups for individual items into a single element. Specific searches were carried out while keeping the initial and final criteria of the string consistent, and adapting it as needed to match the keywords relevant to each topic. The most recent analysis yielded 1,722 results, including 559 articles on airway management, 117 on vascular access, 298 on pain management, 394 on bleeding control, and 258 on devices and equipment. The general criteria included only studies on adult humans, published after the year 2000 in English, with full-text access whenever possible. In this edition, emerging fields such as microgravity and space medicine were excluded, as the Panel lacked direct experience in these areas.

(((((((((((((((((((wilderness medicine) OR (“austere”[Title/Abstract] AND (“medicine”[MeSH Terms] OR “medicine”[All Fields] OR “medicines”[All Fields]))) OR ((“tactic”[Title/Abstract] OR “tactical”[Title/Abstract] OR “tactically”[Title/Abstract]) AND (“medicine”[MeSH Terms] OR “medicine”[All Fields]))) OR (“wild”[Title/Abstract] AND (“medicine”[MeSH Terms] OR “medicine”[All Fields]))) OR ((“remote”[Title/Abstract] OR “remotely”[Title/Abstract] OR “remoteness”[Title/Abstract] OR “remotes”[Title/ Abstract]) AND (“medicine”[MeSH Terms] OR “medicine”[All Fields]))) OR ((“mountain”[Title/Abstract] OR “mountaineer”[Title/ Abstract] OR “mountaineering”[MeSH Terms] OR “mountaineering”[Title/Abstract] OR “mountaineers”[Title/Abstract] OR “mountainous”[Title/Abstract] OR “mountains”[Title/Abstract]) AND (“medicine”[MeSH Terms] OR “medicine”[All Fields]))) OR ((“polar”[Title/Abstract] OR “polars”[Title/Abstract]) AND (“medicine”[MeSH Terms] OR “medicine”[All Fields]))) OR ((“ocean”[Title/ Abstract] OR “ocean s”[Title/Abstract] OR “oceanic”[Title/Abstract] OR “oceans and seas”[MeSH Terms] OR (“oceans”[Title/ Abstract] AND “seas”[Title/Abstract]) OR “oceans and seas”[Title/Abstract] OR “oceans”[Title/Abstract]) AND (“medicine”[MeSH Terms] OR “medicine”[All Fields]))) OR ((“jungle”[Title/Abstract] OR “jungles”[Title/Abstract]) AND (“medicine”[MeSH Terms] OR “medicine”[All Fields]))) OR ((“extreme”[Title/Abstract] OR “extremes”[Title/Abstract]) AND (“medicine”[MeSH Terms] OR “medicine”[All Fields]))) OR ((“expeditions”[MeSH Terms] OR “expeditions”[Title/Abstract] OR “expedition”[Title/Abstract]) AND (“medicine”[MeSH Terms] OR “medicine”[All Fields]))) OR ((“desert”[Title/Abstract] OR “desertic”[Title/Abstract] OR “deserts”[Title/Abstract]) AND (“medicine”[MeSH Terms] OR “medicine”[All Fields]))) OR ((“arctic regions”[MeSH Terms] OR (“arctic”[Title/Abstract] AND “regions”[All Fields]) OR “arctic regions”[Title/Abstract] OR “arctic”[Title/Abstract]) AND (“medicine”[MeSH Terms] OR “medicine”[All Fields]))) OR ((“antarctic regions”[MeSH Terms] OR (“antarctic”[Title/Abstract] AND “regions”[All Fields]) OR “antarctic regions”[Title/Abstract] OR “antarctic”[Title/Abstract]) AND (“medicine”[MeSH Terms] OR “medicine”[All Fields]))) OR (military medicine)) OR (“search and rescue”[Title/Abstract] OR “SAR”[Title/Abstract])) OR ((“air ambulances”[MeSH Terms] OR (“air”[Title/Abstract] AND “ambulances”[Title/Abstract]) OR “air ambulances”[Title/Abstract] OR (“helicopter”[Title/Abstract] AND “emergency”[Title/Abstract]) OR “helicopter emergency”[Title/Abstract]) AND (“medic”[All Fields] OR “medical”[All Fields] OR “medicalization”[MeSH Terms] OR “medicalization”[All Fields] OR “medicalizations”[All Fields] OR “medicalize”[All Fields] OR “medicalized”[All Fields] OR “medicalizes”[All Fields] OR “medicalizing”[All Fields] OR “medically”[All Fields] OR “medicals”[All Fields] OR “medics”[All Fields]) AND (“system”[All Fields] OR “system s”[All Fields] OR “systems”[All Fields]))) OR (disaster medicine)))

AND

((((“rapid sequence induction and intubation”[MeSH Terms] OR (“rapid”[Title/Abstract] AND “sequence”[Title/Abstract] AND “induction”[Title/Abstract] AND “intubation”[Title/Abstract]) OR “rapid sequence induction and intubation”[Title/Abstract] OR (“rapid”[Title/Abstract] AND “sequence”[Title/Abstract] AND “induction”[Title/Abstract]) OR “rapid sequence induction”[Title/ Abstract] OR “RSI” [Title/Abstract])) OR (cricothyrotomy) OR (cricothyroidotomy) OR (endotracheal tube) OR (Videolaryngoscope) OR (glidescope) OR (cmac) OR (video laryngoscopy) OR (Laryngeal mask) OR (Supraglottic devices) OR (airways management))

OR

(((“injections, intravenous”[MeSH Terms] OR (“injections”[All Fields] AND “intravenous”[All Fields])) OR “intravenous injections”[All Fields] OR “injections intravenous”[All Fields]) OR ((“blood vessels”[MeSH Terms] OR “vascular”[Title/Abstract]) AND (“access”[Title/Abstract] OR “accesses”[Title/Abstract] OR “accessibilities”[Title/Abstract] OR “accessibility”[Title/Abstract] OR “accessible”[Title/Abstract] OR “accessing”[Title/Abstract])) OR ((“bone and bones”[MeSH Terms] OR (“bone”[Title/Abstract] AND “bones”[Title/Abstract])) AND (“inject”[Title/Abstract] OR “injectant”[Title/Abstract] OR “injectants”[Title/Abstract] OR “injectate”[Title/Abstract] OR “injectates”[Title/Abstract] OR “injected”[Title/Abstract] OR “injectible”[Title/Abstract] OR “injectibles”[Title/Abstract] OR “injecting”[Title/Abstract] OR “injections”[Title/Abstract] OR “injectable”[Title/Abstract] OR “injectables”[Title/Abstract] OR “injection”[Title/Abstract] OR “injects”[Title/Abstract])) OR ((“infusions, intraosseous”[MeSH Terms] OR (“infusions”[Title/Abstract] AND “intraosseous”[Title/Abstract]) OR “intraosseous infusions”[Title/Abstract] OR “infusions intraosseous”[Title/Abstract])) OR ((“central”[Title/Abstract] OR “centrally”[Title/Abstract] OR “centrals”[Title/Abstract]) AND (“veins”[MeSH Terms] OR “veins”[Title/Abstract] OR “venous”[Title/Abstract]) AND (“access”[Title/Abstract] OR “accessed”[Title/Abstract] OR “accesses”[Title/Abstract] OR “accessibilities”[Title/Abstract] OR “accessibility”[Title/Abstract] OR “accessible”[Title/Abstract] OR “accessing”[Title/Abstract])) OR ((“peripheral”[Title/Abstract] OR “peripherally”[Title/Abstract] OR “peripherals”[Title/Abstract] OR “periphereal”[Title/Abstract] OR “peripheric”[Title/Abstract] OR “peripherically”[Title/ Abstract]) AND (“veins”[MeSH Terms] OR “veins”[Title/Abstract] OR “venous”[Title/Abstract]) AND (“access”[Title/Abstract] OR “accessed”[Title/Abstract] OR “accesses”[Title/Abstract] OR “accessibilities”[Title/Abstract] OR “accessibility”[Title/ Abstract] OR “accessible”[Title/Abstract] OR “accessing”[Title/Abstract])) OR ((“central venous catheters”[MeSH Terms] OR (“central”[Title/Abstract] AND “venous”[Title/Abstract] AND “catheters”[Title/Abstract]) OR “central venous catheters”[Title/ Abstract] OR (“central”[Title/Abstract] AND “venous”[Title/Abstract] AND “catheter”[Title/Abstract]) OR “central venous catheter”[Title/Abstract])) OR ((“peripheral”[Title/Abstract] OR “peripherally”[Title/Abstract] OR “peripherals”[Title/Abstract] OR “periphereal”[Title/Abstract] OR “peripheric”[Title/Abstract] OR “peripherically”[Title/Abstract]) AND (“veins”[MeSH Terms] OR “veins”[Title/Abstract] OR “venous”[Title/Abstract]) AND (“catheter s”[Title/Abstract] OR “catheters”[MeSH Terms] OR “catheters”[Title/Abstract] OR “catheter”[Title/Abstract])))

OR

((“pain”[MeSH Terms]) AND ((“ketamine”[Title/Abstract]) OR (“ketofol”[Title/Abstract]) OR (“morphine”[Title/Abstract]) OR (“fentanyl”[Title/Abstract]) OR (“opioids”[Title/Abstract]) OR (“loco regional anesthesia”[Title/Abstract]) OR (“regional anesthesia”[Title/Abstract]) OR (“peripheral nerve block”[Title/Abstract]) OR (“pain scale”[Title/Abstract]) OR (“analgesia”[Title/ Abstract]) OR “pain guideline”[Title/Abstract]) OR (“pain management”[Title/Abstract]))

OR

(((“hemorrhage”[MeSH Terms) OR (“blood coagulation”[MeSH Terms) OR (“tourniquets”[MeSH Terms) OR (“acidosis”[MeSH Terms)) AND ((“fibrinogen”[Title/Abstract]) OR (“tranexamic”[Title/Abstract]) OR (“calcium”[Title/Abstract]) OR (“hypothermia”[Title/Abstract]) OR (“packing”[Title/Abstract]) OR (“combat gauze”[Title/Abstract]) OR (“bleeding”[Title/ Abstract]) OR (“shock hemorrhagic” [Title/Abstract]) OR (“massive bleeding”[Title/Abstract]) OR (“stop the bleed”[Title/ Abstract])))

OR

(((“equipment and supplies”[MeSH Terms) OR (“oxygen”[MeSH Terms) OR (“ultrasonography”[MeSH Terms])) AND ((“oxygen concentrator”[Title/Abstract]) OR (“echography” [Title/Abstract]) OR (“point of care”[Title/Abstract]) OR (“3D printing”[Title/ Abstract]) OR (“comunication”[Title/Abstract]) OR (“solar power”[Title/Abstract]) OR (“telemedicine”[Title/Abstract]) OR (“teleconsulting”[Title/Abstract]) OR (“telementoring” [Title/Abstract]) OR (“traning”[Title/Abstract]) OR (“education”[Title/ Abstract]))))

AND

(((english[Filter]) AND (alladult[Filter]) AND (2000:2024[pdat])) AND ((english[Filter]) AND (alladult[Filter])) AND ((english[Filter]) AND (alladult[Filter]) AND (2000:2024[pdat])))
