# Supplementary material for: Management of critically ill patients in austere environments: good clinical practice by the Italian Society of Anesthesia, Analgesia, Resuscitation and Intensive Care (SIAARTI)
Source: J Anesth Analg Crit Care. 2024 Nov 6;4:74. doi: 10.1186/s44158-024-00209-8 (PMC11542215; doi:10.1186/s44158-024-00209-8)
Supplement: Supplementary file 2 — Supplementary Material 2 [file 44158_2024_209_MOESM2_ESM.docx]

**Management of Critically Ill Patients in Austere Environments: Good Clinical Practice by the Italian Society of Anesthesia, Analgesia, Resuscitation and Intensive Care (SIAARTI**

**APPENDIX A - FLOWCHART**

Rapid Sequence Intubation (RSI): The 8 Ps

**PREPARATION**

Team:

- Designate the team leader
- Assign roles
- Develop and communicate the intubation plan

Equipment (SOAP-ME):

- Suction
- Oxygen
- Airway management tools
- Pharmacology
- Monitoring Equipment

Patient (LEMON):

- Look externally
- Evaluate 3-3-2
- Mallampati score
- Obstruction
- Neck mobility
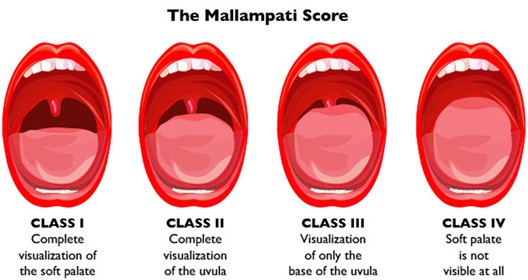


**Prediction of Difficult Mask Ventilation:**

- Mallampati class 4
- Obstructive sleep apnea (OSA) (history of snoring, Stop Bang Scoring Model)
- Obesity (BMI > 26 kg/m²)
- Prominent nasal structure
- Edentulism (toothlessness)
- Macroglossia (enlarged tongue)
- Beard
- Age > 55 years

**Prediction of Difficult Intubation by Single Parameter:**

- Mallampati class 4
- Thyromental distance < 6 cm
- Inter-incisor distance < 3 cm (intubation is impossible if < 2 cm)
- Cervical spine rigidity (limited flexion)
- Maxillary prognathism > 1 cm (uncompensated by mandibular subluxation)

**Prediction of Difficult Intubation by Multiple Parameters:**

- Mallampati class 3
- Thyromental distance 6-6.5 cm
- Inter-incisor distance 3-3.5 cm
- Reduced neck mobility
- Maxillary prognathism compensated by mandibular subluxation

**Prediction of Difficulty Using Extra-Glottic Devices (EGD):**

- Mouth opening < 2 cm
- Edema at the glottic inlet
- Anatomical abnormalities of the oropharynx
- High risk of aspiration

**PREOXYGENATION**

**Methods:**

- **Slow:** Normal tidal breathing for 3 minutes with FiO₂ 100%
- **Fast:** 4-8 deep breaths over 30-60 seconds with FiO₂ 100%
- **NIV/CPAP:** Consider for patients with reduced DAWD (Duration of Apnea Without Desaturation)

**DAWD = Duration of Apnea without Desaturation**

**(Desaturation defined as SpO2 < 90%, healthy individual 5-6 minutes)**

**Patients with Reduced DAWD (1-3 minutes):**

- Obesity
- OSA
- COPD
- Upper airway infections
- ASA classification 3-4
- Extremes of age (elderly or pediatric patients)
- Pregnant patients
- Critically ill patients (ascites, abdominal compartment syndrome, etc.)

**PRIMING**

- Midazolam 0.02mg/kg
- Fentanyl 1-2 mcg/kg
- 500 ml crystalloid IV, IO (except in patients with CHF)

**PRIME**

- - Propofol 2.5 mg/kg titrated
  - Ketamine 3-5 mg/kg
  - Midazolam 0.2mg/kg
  - Etomidate 0.3 mg/kg

**PRESSURE**

- Sellick maneuver

**PARALYSIS**

- Rocuronium 1.2 mg/kg
- Succinylcholine 1-1.5 mg/kg

**Contraindications to Succinylcholine:**

- Allergy
- Hyperkalemia
- Severe acidosis
- Neuromuscular disease (acute and chronic)
- Burn victim (more than 48 hours)
- Spinal trauma

**POSITIONING**

- Visualization of the larynx
- Advancement of the endotracheal tube
- Introduction of the endotracheal tube

**1st ATTEMPT**

Difficulty visualizing:

- Request help from a colleague (if available)
- Reposition the head

Attention: Maintain the head-neck-trunk axis in trauma

- Manipulate the larynx

OELM (Optimal External Laryngeal Manipulation)

BURP (Backward, Upward, Rightward Pressure)

- Monitor vital signs (A, B, C, D, E)

Assessment:

- - Degree of visualization difficulty (Cormack-Lehane modified by Cook)
  - Mask ventilation ability (deterioration in ventilability)

Action:

- Ensure the patient’s ventilation and oxygenation

**2nd ATTEMPT**

**Difficulty in visualization:**
✓ Increased traction on the laryngoscope
✓ Repositioning of the head
**Attention:** Maintain the head-neck-torso alignment in trauma cases
✓ Laryngeal manipulation
OELM (Optimal External Laryngeal Manipulation)
BURP (Backward, Upward, Rightward Pressure)
✓ Monitoring of vital signs (A, B, C, D, E)

**Assessment:**
✓ Degree of visualization difficulty (Cormack-Lehane modified by Cook)
✓ Mask ventilation ability (deterioration in ventilability)

**Action:**
✓ Decide the strategy based on the clinical evaluation and visualization during direct laryngoscopy


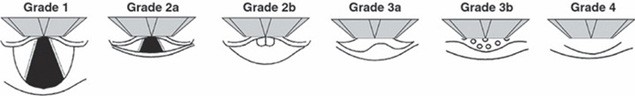


**Cormack 1 and 2a:** Curved blade laryngoscope (Macintosh), stylet-guided tube
**Cormack 2b:** Curved blade laryngoscope (Macintosh) with Frova introducer
**Cormack 3a and 3b:** Videolaryngoscopy
**Cormack 4:** Fiberoptic intubation through a laryngeal mask airway

**3rd ATTEMPT**

✓ Avoid repeated attempts without changing the technique and/or equipment
✓ Stay focused on the objective: Ventilation and Oxygenation
✓ Ensure that ventilability does not deteriorate

**4th ATTEMPT**

**INSERT AN EGD (Extraglottic Device):**
✓ Second generation (with the option for gastric tube insertion)
✓ Intubatable (with the option for bronchoscope-guided intubation through the EGD)

**If the patient is ventilable:**
✓ Ensure the patient’s ventilation and oxygenation
✓ Monitoring of vital signs (A, B, C, D, E)
✓ Consider bronchoscope-guided intubation through the EGD (if available)

**If the patient is not ventilable (CICO - Cannot Intubate, Cannot Oxygenate):**
✓ Perform emergency cricothyroidotomy (use a cricothyroidotomy kit)
✓ Ensure oxygenation of the patient via jet ventilation (if available)
✓ Monitoring of vital signs (A, B, C, D, E)
✓ Call an ENT surgeon (if available) for surgical airway in emergency/urgent cases

**POST-INTUBATION**

Continuous sedation-analgesia for the patient
**Tube position verification:**
• Auscultation
✓ Apices
✓ Bases
✓ Epigastrium
• Aspiration with a 50 ml wide-bore syringe
• Self-expanding bulb (Oesophage Detection Device)
• Capnographic waveforms
✓ At least five consecutive
✓ Consistent morphology
• Direct visualization with a bronchoscope (if available and necessary)
